# Chitosan/reduced graphene oxide/papain nanocomposite: a bioactive platform for skincare and biomedical applications

**DOI:** 10.1038/s41598-026-49660-3

**Published:** 2026-04-20

**Authors:** Niloufar Elhami, Mohammad Pazhang

**Affiliations:** Department of Biology, Faculty of Sciences, Azarbaijan Shahid Madani University, Tabriz, Iran

**Keywords:** Chitosan, Reduced graphene oxide, Papain, Wound healing, Skin care, Biotechnology, Chemistry, Materials science, Nanoscience and technology

## Abstract

This work introduces a bioactive chitosan-based nanocomposite incorporating reduced graphene oxide and papain (CS/rGO/Pa), designed for wound healing and cosmetic facial mask applications. The nanocomposite was fabricated via ionic gelation and characterized by Fourier Transform Infrared (FTIR), X-ray diffraction (XRD), Scanning Electron Microscope (SEM) and Differential Light Scattering (DLS). Subsequently, the porosity, swelling, degradation of CS/rGO/Pa and papain release/activity was evaluated. In vitro assays assessed its anti-oxidant, anti-inflammatory, anti-bacterial, and proliferation properties. The results of FTIR, XRD, SEM and DLS indicated that the nanoparticles were synthesized correctly with an approximately spherical morphology, an average size of around 100 nm, a hydrodynamic diameter of 553 nm, and zeta potential of − 2.97 mV. The results showed that adsorption of papain on CS/rGO decreased papain activity from 7.3 to 0.57 U/mg which can be favorable for wounded epithelial tissues and skin care applications, requiring low and sustained enzymatic action. The release rate results indicated a higher release rate in a basic environment (pH 8.4), and the Korsmeyer–Peppas model for the release of papain. The results of CS/rGO/Pa properties indicated proper porosity, swelling and degradation, with 93% anti-inflammatory, 20% anti-oxidant activity, high anti-bacterial effect, and cell growth up to 7.91-fold for 7 days. In conclusion, the outcomes represented that the CS/rGO/Pa nanocomposite can be a favorable and effective material for using in skincare and biomedical applications.

## Introduction

As the body’s largest organ and primary line of defense, the skin serves as a vital barrier against environmental aggressors such as ultraviolet (UV) radiation, airborne nanoparticles, and microbial pathogens^1,2^. Continuous exposure to these factors promotes oxidative damage and inflammation^3^. This has driven increasing interest in cosmetic formulations that not only protect but also actively support skin regeneration through natural and bioactive compounds^4^.

Recent studies on graphene-based systems have demonstrated that engineering graphene into functional composites can enable simultaneous improvements in sensitivity, mechanical performance, and biocompatibility, depending on the host matrix and application. In particular, graphene has been shown to play a key role in advanced healthcare technologies, ranging from disease monitoring platforms to reinforced and load-bearing biomedical composites, where interfacial interactions govern performance. These observations emphasize the role of synergistic material design and further motivate the combination of graphene with biopolymers such as chitosan for multifunctional bioactive systems^5–7^.

Chitosan (CS) is a highly attractive carrier for enzyme immobilization in biomedical applications, drug formulation, and delivery, owing to its excellent biocompatibility, biodegradability, non-toxicity, and anti-microbial^8–11^. Chitosan is a favorable carrier for enzyme immobilization due to its structure^8^. Papain (Pa) is a highly thermostable cysteine protease^12^ and one of the most extensively studied proteolytic enzymes for exfoliation. It exhibits a range of biological activities, including anti-inflammatory, anti-bacterial, anti-oxidant effects, as well as the debridement^13,14^. Reduced graphene oxide (rGO), due to its high surface area and structural versatility, enhances the mechanical strength of composite systems, while also exhibiting significant anti-bacterial, and cytotoxic activities^11,15–17^. However, despite their established roles in wound healing, the integration of these materials into facial mask formulations remains unexplored, representing a novel approach investigated in this study. To the best of our knowledge, this is the first study to develop a cosmetic facial mask based on a papain immobilized chitosan/reduced graphene oxide nanocomposite, designed to simultaneously provide exfoliation, enhanced enzyme stability, and multifunctional bioactivity.

A major challenge in enzyme-based formulations is maintaining activity over time and in complex environments. Immobilizing enzymes on nanocarriers like chitosan or graphene-based materials can enhance their stability, protect against denaturation, controlled activity, and provide controlled release^15,18–21^. This study investigates the use of a CS/rGO nanocomposite as a dual-purpose matrix for enzyme immobilization (by adsorption) and bioactive delivery in cosmetic facial masks. The nanocomposite was prepared using the ion gelation method and characterized by Fourier Transform Infrared (FTIR), Differential Light Scattering (DLS), and X-ray diffraction (XRD) techniques. Finally, papain immobilized on the matrix by adsorption and its activity were assessed, along with the formulation’s anti-inflammatory, anti-oxidant, anti-bacterial, and cell proliferation properties through in vitro experiments.

## Materials and methods

### Materials

Chitosan (MW: 50–190 kDa; ≥ 75% deacetylation), reduced graphene oxide, papain, sodium tripolyphosphate (TPP), acetic acid, sodium phosphate, ethanol, methanol, sodium chloride, sodium citrate, dextrose, citric acid, and nutrient agar were obtained from Merck (Germany). DMEM-RPMI1640, Hydrate 2,2-diphenyl-2- picrylhydrazyl, dimethyl sulfoxide (DMSO), 3-(4,5-dimethylthiazol-2-yl)-2,5-diphenyltetrazolium bromide (MTT), trypsin, and Penicillin-G were bought from Sigma (USA). Fetal bovine serum (FBS) was achieved from Azar Zist Farayand (Iran).

### CS/rGO/Pa nanocomposite synthesis

The ion gelation method was employed to prepare the chitosan nanoparticles (CSNP) and CS/rGO nanocomposite^22^. CSNPs were prepared by dissolving 50 mg chitosan in 5 mL acetic acid, followed by dropwise addition of 10% TPP (TPP/CS: 5% v/v). The mixture was centrifuged (10000 rpm, 1 min), rinsed three times with distilled water, and stored in phosphate buffer for later use.

To obtain CS/rGO, 50 mg of chitosan dissolved in 5 mL acetic acid. In an ultrasonic bath, 0.625 mg/mL rGO suspended in ethanol and the suspension was added dropwise to the chitosan solution. Subsequently, TPP solution (10% w/v) (TPP/CS ratio of 5% v/v) was added dropwise to the CS/rGO solution and centrifuged (10000 rpm, 1 min). Next, the CS/rGO patch was rinsed three times with distilled water and completely dissolved in a 5 mL phosphate buffer solution (pH 7.4, 50 mM).

Finally, to adsorb the enzyme on the nanocomposites and obtain CS/rGO/Pa, 1 mL of 15 mg/mL of papain dissolved in sodium phosphate buffer solution. The papain solution was added dropwise to the CS/rGO nanocomposite solution on a stirrer at 4 °C for 4 h, and then centrifuged (10000 rpm, 2 min). The supernatant was transferred into a new tube and kept for subsequent tests, and the CS/rGO/Pa patch was rinsed three times and kept in sodium phosphate buffer for the later steps. Papain stability was evaluated under the adsorption conditions (4 °C, 4 h), and only negligible changes in enzymatic activity were observed (result not showed).

To standardize patch size and thickness and ensure comparability, all CS/rGO/Pa patches were prepared using fixed-dimension molds. Each mold received the same volume of solution, ensuring equal weight and contact area. Patches were then dried under uniform conditions, giving identical thickness and surface contact. This standardization allows meaningful comparison of subsequent measurements.

The quantity of papain adsorbed on the CS/rGO matrix, as well as that remaining in the supernatant was quantified spectrophotometrically using an ultraviolet-visible spectrophotometer at the maximum wavelength of 595 nm (with the Bradford method)^23^. The immobilization efficiency was calculated using Eq. (1).

1 $$Immobilization\,\,Yield\% \, = \,\left[ {\left( {Abs_{{total\,\,enzyme}} {-}{\text{ }}Abs_{{supernatant}} } \right){\mathrm{/}}Abs_{{total\,\,enzyme}} } \right] \times 100$$

### Fabrication of wound dressing in cotton for anti-bacterial and cell culture analysis

To achieve a CS/rGO/Pa patch for anti-bacterial and cell culture analysis, natural cotton fabric was utilized. Cotton is natural, biodegradable, highly absorbent, and breathable. It promotes a dry environment and can help absorb exudate while limiting bacterial growth^24–27^. Overall, cotton dressings offer anti-microbial benefits for wound care. Therefore, the cotton fabric was cut into 1.5 × 1.5 cm squares and autoclaved. Under sterile conditions, these cottons were immersed in 100 µL of nanocomposite solution at a concentration of 16 mg/mL. Finally, the samples were designated as CSNP and CS/rGO/Pa.

### Characterization of CS/rGO/Pa

Fourier Transform Infrared spectrometry (FTIR) analysis of CS/rGO/Pa was performed to characterize the functional groups present in the samples. It was carried out using the Bruker FT-IR spectrometry (VECTOR22, Germany) in the wave range from 400 to 4000 cm^− 1^.

Dynamic Light Scattering (DLS) analysis was conducted to determine the nanocomposite solution’s hydrodynamic dimension and zeta potential, utilizing a blue LAB DLS apparatus from Malvern, England.

X-ray diffraction (XRD) was employed to validate the crystallinity of the nanocomposites, utilizing a GNR XRD instrument (Theta/Theta XRD – Explorer, Italy) with operational parameters set at a current of 35 mA, voltage of 40 kV, and scanning range of 10–70.0°.

The surface morphology of the nanocomposite (CS/rGO/Pa) was examined by Field Emission Scanning Electron Microscopy (FESEM) using a TESCAN MIRA III (Czech Republic) operated at an accelerating voltage of 15 kV.

### Enzyme activity measurement

The papain activity was determined using casein as a substrate^28^. In this procedure, 100 µL of the enzyme solution (the free or immobilized enzyme dissolved in 50 mM phosphate buffer, pH 7.4) was added to 400 µL of phosphate buffer (50 mM, pH 7.4) containing casein (1% v/w). After, incubation for 1 h at 25 °C, 500 µl trichloroacetic acid (TCA) (10% w/v) was added to the mixture to stop the reaction. Afterward, the samples were centrifuged (10000 rpm, 10 min) and the supernatant absorbance was analyzed by at 280 nm. One unit of activity (U) was considered as the amount of enzyme capable of reacting with casein and producing an absorbance equivalent to 1 µmol of tyrosine/min.

### Analysis of the papain-release kinetics

In order to determine papain-release rate^23^, 160 mg of CS/rGO/Pa patch was placed in 5 mL phosphate buffer solution (pH 6.4, 7.4, 8.4, 50 mM) at 37 °C with shaking (180 rpm, 48 h). Every 4 h sampling was done by removing 0.1 mL from the solutions and replacing it with 0.1 mL of fresh sodium phosphate buffer (with the appropriate pH). Then the samples were centrifuged (10000 rpm, 2 min), and analyzed by Bradford assay at 595 nm by an UV spectrophotometer. The bovine serum albumin was utilized as standard. The kinetics of drug release were analyzed using various mathematical models, including the zero-order (Eq. 2), first-order (Eq. 3), Higuchi (Eq. 4), and Korsmeyer–Peppas (Eq. 5) equations, facilitated by the DDSolver.xla extension in Microsoft Excel^29–32^.

2 $$Q_{t} = k_{0} \times t + Q_{0}$$

3 $$Q_{t} = \left( {1{-}e^{{ - kt}} } \right) \times Q_{0}$$

4 $$Q_{t} = k_{H} {-}\surd t$$

5 $$Q_{t} = k \times t^{n} \times Q_{0}$$

### Porosity test

Ethanol was used as a displacement liquid to analyze patch porosity. CS/rGO/Pa patch was placed in 5 mL ethanol (v_1_), and its volume alteration was recorded (v_2_). Subsequently, the patch was removed by centrifuging (1000 rpm, 2 min) after 2 h, and the second alteration of ethanol volume was recorded (v_3_). To evaluate the porosity, the following equation Eq. 6 was used^33^.

6 $$Porosity\% = \left( {v_{1} - v_{3} } \right)/\left( {v_{2} - v_{3} } \right) \times 100$$

### Swelling and degradation test

The swelling and degradation rate were determined using sodium phosphate buffer solution (pH 7.4, 50 mM) at 37 °C for 96 h. To assess the swelling rate, firstly, the CS/rGO/Pa patch was weighed (w _dry1_), placed in the buffer, and incubated. Then after a specific time (24, 48, 72, and 96 h), the patch was extracted and weighed (w _wet_).

To assess the degradation rate, first, the CS/rGO/Pa was weighed (w _dry1_), placed in the buffer, and incubated. Then after a specific time (24, 48, 72, and 96 h), the patch was extracted, dried, and weighed (w _dry2_). Finally, the following equations were used to obtain the swelling (Eq. 7) and degradation (Eq. 8) rate^33^.

7 $$Swelling\,\,rate\% = \left( {w_{{wet}} {-}{\text{ }}w_{{dry1}} } \right)/w_{{dry1}} \times 100$$

8 $$Degradation\,\,rate\% = \left( {w_{{dry1}} {-}{\text{ }}w_{{dry2}} } \right)/w_{{dry1}} \times 100$$

### In vitro anti-inflammatory test

In order to assess the anti-inflammatory efficiency, a stabilization method was employed. Human blood samples were obtained from healthy human volunteers. All experiments involving human blood were carried out in accordance with relevant guidelines and regulations and were approved by the Ethics Committee of Azarbaijan Shahid Madani University (Tabriz, Iran), with approval number of IR. AZARUNIV.REC.1402.006. The informed consent was obtained from all subjects. Initially, 1 mL Alsever’s solution (2% dextrose, 0.8% sodium citrate, 0.5% citric acid, and 0.42% sodium chloride) was added to 1 mL whole blood and centrifuged (1000 rpm, 10 min). The supernatant was then removed, and the red blood cell (RBC) pellets were rinsed three times with isotonic saline. Next, 250 µL RBC, 500 µL sodium phosphate buffer solution (pH 7.4, 50 mM), and 1 mL hypo-saline were added to 200 µL of prepared various concentrations (2–16 mg/mL) of CS/rGO/Pa dissolved in distilled water. The suspension was incubated for 30 min at 37 °C, centrifuged (3000 rpm, 5 min), and supernatants were collected and measured at 540 nm. The control was 250 µL RBC, 500 µL sodium phosphate buffer solution (pH 7.4, 50 mM), and 1 mL hypo saline solution. Finally, the following equation Eq. 9 was used to obtain the stabilization^22^.

9 $$Stabilization\% = 100 - \left( {Abs_{{sample}} /Abs_{{control}} \times 100} \right)$$

### Anti-oxidant test

2,2-diphenyl-2-picrylhydrazyl (DPPH) was employed to evaluate the anti-oxidant efficiency. In detail, 500 µL of 400 ppm of CS/rGO/Pa dissolved in methanol was added to 500 µL of 0.08 mg/mL of DPPH dissolved in methanol and incubated in a dark place (25 °C, 30 min). Subsequently, the sample was centrifuged (10000 rpm, 5 min), supernatant was collected and measured at 517 nm. The control was 500 µL DPPH solution and 500 µL methanol. The following equation Eq. 10 was used to obtain the anti-oxidant activity^34^.

10 $$Anti{\text{ - }}oxidant\% = \left[ {\left( {Abs_{{control}} {-}Abs_{{sample}} } \right)/Abs_{{control}} } \right] \times 100$$

### Anti-bacterial test

In this test, disk diffusion method was employed and performed against *Staphylococcus aureus* and *Pseudomonas aeruginosa* on nutrient agar plates. The CS/rGO/Pa was loaded onto cotton, and placed on the culture medium. The control was a plain cotton due to its intrinsic anti-bacterial properties, allowing for a more accurate assessment of the nanocomposite’s specific effects^24,26^. The plates were incubated (37 °C, 18 h), and the diameter of the haloes was measured^33^.

### Cell culture and proliferation test

In order to perform the MTT assay, the NIH-3T3 cell line, as a well-established and widely recognized fibroblast model for wound healing and tissue regeneration studies^2,33^, was obtained from the Pasteur Institute of Iran and cultured in RPMI 1640 medium supplemented with 10% FBS and 1% Penicillin-G. The cells were incubated (5% CO_2_, 37 °C, 3, 5, and 7 days) to assess cell growth. The initial density of cultured cells in 24-well plates was at 1 × 10^5^ cells/mL, containing sterile samples, with 1 mL of medium per well. The control consisted of cells cultured without any material (cells only), representing normal viability. Cotton was used as a reference control due to its known biocompatibility and influence on cell proliferation, enabling a more accurate comparison of the nanocomposite’s effects^25,27^. Following the incubation period, samples were rinsed with phosphate-buffered saline before introducing 10 µL of MTT solution into each well. The plates were subsequently kept in darkness for 4 h. After discarding the solution, 300 µL of DMSO was added to each well and incubated for an additional 2 h. The optical absorbance at 570 nm was then measured to assess cell viability using the specified equation (Eq. 11)^35^.

11 $$Cell\,viability\% = \left( {Abs_{{sample}} /Abs_{{control}} } \right) \times 100$$

### Statistical analysis

Statistical analyses included one-way ANOVA and t-tests to detect significant differences among the data, and measures of model fit (R^2^ and AIC) to evaluate regression and the goodness of fit for the drug-release kinetics. A significance threshold was set at *p < 0.05, with **p < 0.001 indicating a highly significant result.

## Results and discussion

### CS/rGO/Pa preparation and characterization

To evaluate the applicability of the developed nanocomposite for enzyme immobilization and biomedical application such as cosmetic use, various physicochemical and biological properties of the prepared CS/rGO/Pa system were investigated. Papain immobilization yield onto CS/rGO nanocomposite was found to be 53%.

**Fig. 1:**
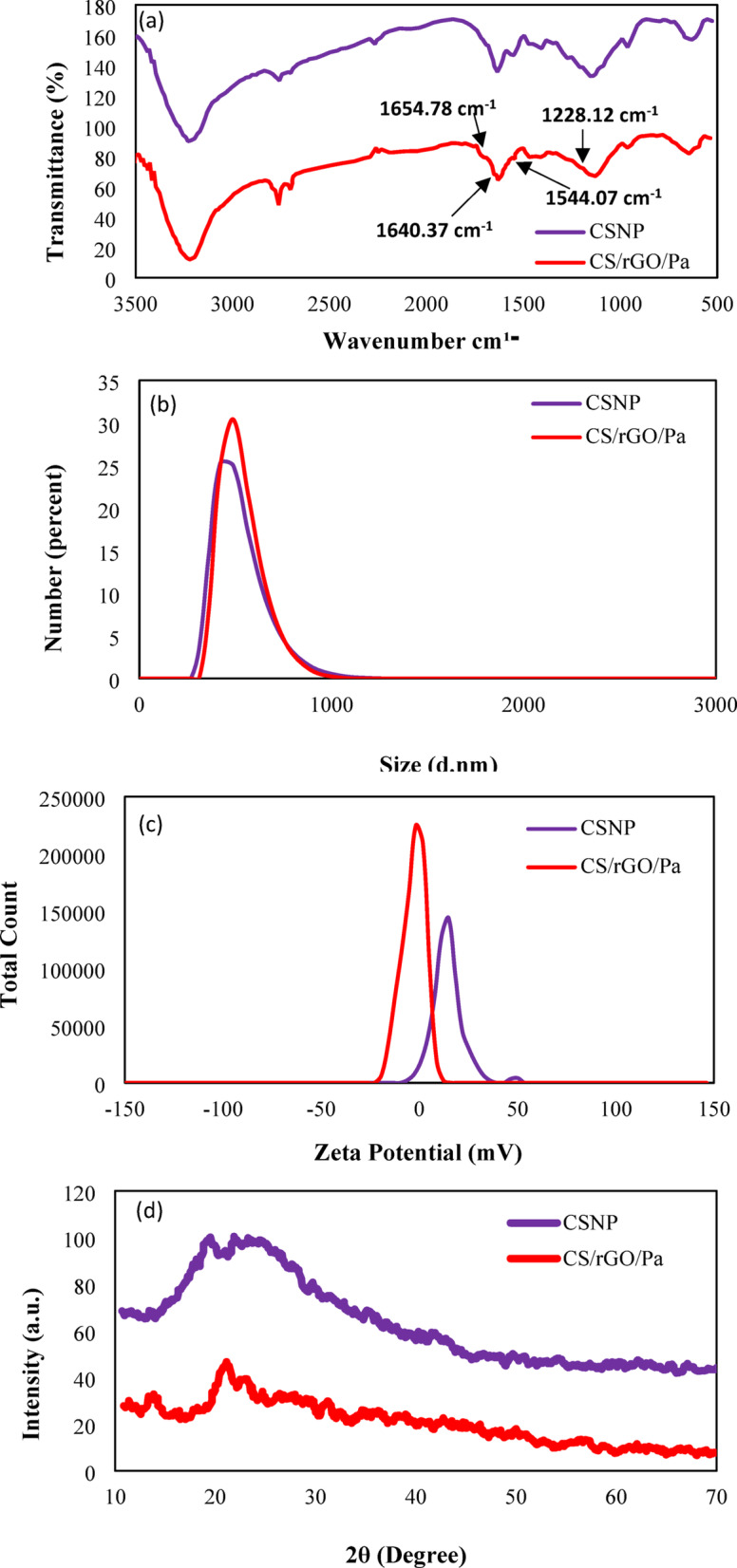
Characterization of CS/rGO/Pa. (**a**) FTIR spectra of CSNP and CS/rGO/Pa, (**b**) DLS: hydrodynamic size of CSNP and CS/rGO/Pa, (**c**) DLS: zeta potential of CSNP and CS/rGO/Pa (**d**) XRD spectra of CSNP, and CS/rGO/Pa.

The chemical composition of CSNP and CS/rGO/Pa is shown in Fig. 1a. Considering the CSNP spectrum, the absorption bands of N–H and O-H partially overlap, extending from 3300 to 3450 cm^−1^, corresponding to the N–H and O–H bonds of chitosan, and in the CS/rGO/Pa spectrum, this band is intensified due to the reduced oxygen groups^36^. Peaks at 1640 and 1228 cm^−1^, corresponding to C–O and C=O bonds respectively, indicate the bonding between chitosan and rGO^11^. The absorption band at 1654 cm^−1^ corresponds to the C–O stretching in the primary amide region, and the absorption band at 1544 cm^−1^ is due to the N–H bending vibration and C-N stretching vibration in the secondary amide region, which is observed in this compound due to the presence of papain^23^.

The results of hydrodynamic size indicated that CSNP and CS/rGO/Pa assemble into particles with diameters of 490 nm and 553 nm, respectively, (Table 1) because of their charge and amphipathic nature. Generally, the hydrodynamic size increases with the addition of papain and rGO (Table 1; Fig. 1b). Zeta potential was recorded at 14.85 mV for CSNP and − 2.97 mV for CS/rGO/Pa. The zeta potential results show that incorporating rGO and papain altered the sample’s surface charge. Chitosan is generally positively charged under acidic conditions due to protonation of its amino groups (–NH2), which also occurs around physiological pH. At higher pH, these groups may deprotonate, leading to reduced positive charge^37^. Papain molecules may carry negatively charged groups, which can interact with the positively charged chitosan nanoparticles, causing a relative decrease in the zeta potential compared to CSNPs^38,39^. Considering the negative charge of rGO^40^, compositing it with CS results in a negative charge of CS/rGO/Pa (Table 1; Fig. 1c).

**Table 1:** Particle diameter and zeta potential of the nanocomposites.

Sample	Particle diameter (nm)	Zeta potential (mV)
CSNP	490	14.85
CS/rGO/Pa	553	− 2.97

The crystal structure of the sample was measured by using XRD. As shown in Fig. 1d, because of crystalline rGO in nanocomposite the peak at 2θ = 27°^41^ has emerged, indicating its successful incorporation into the chitosan matrix. The peak at 2θ = 13° and 19° corresponds to papain^42,43^, confirming its presence within the composite. The broadening and slight shifts of these peaks suggest interactions between chitosan, rGO, and papain, which may alter the crystallinity and ordering of the polymer matrix.

**Fig. 2:**
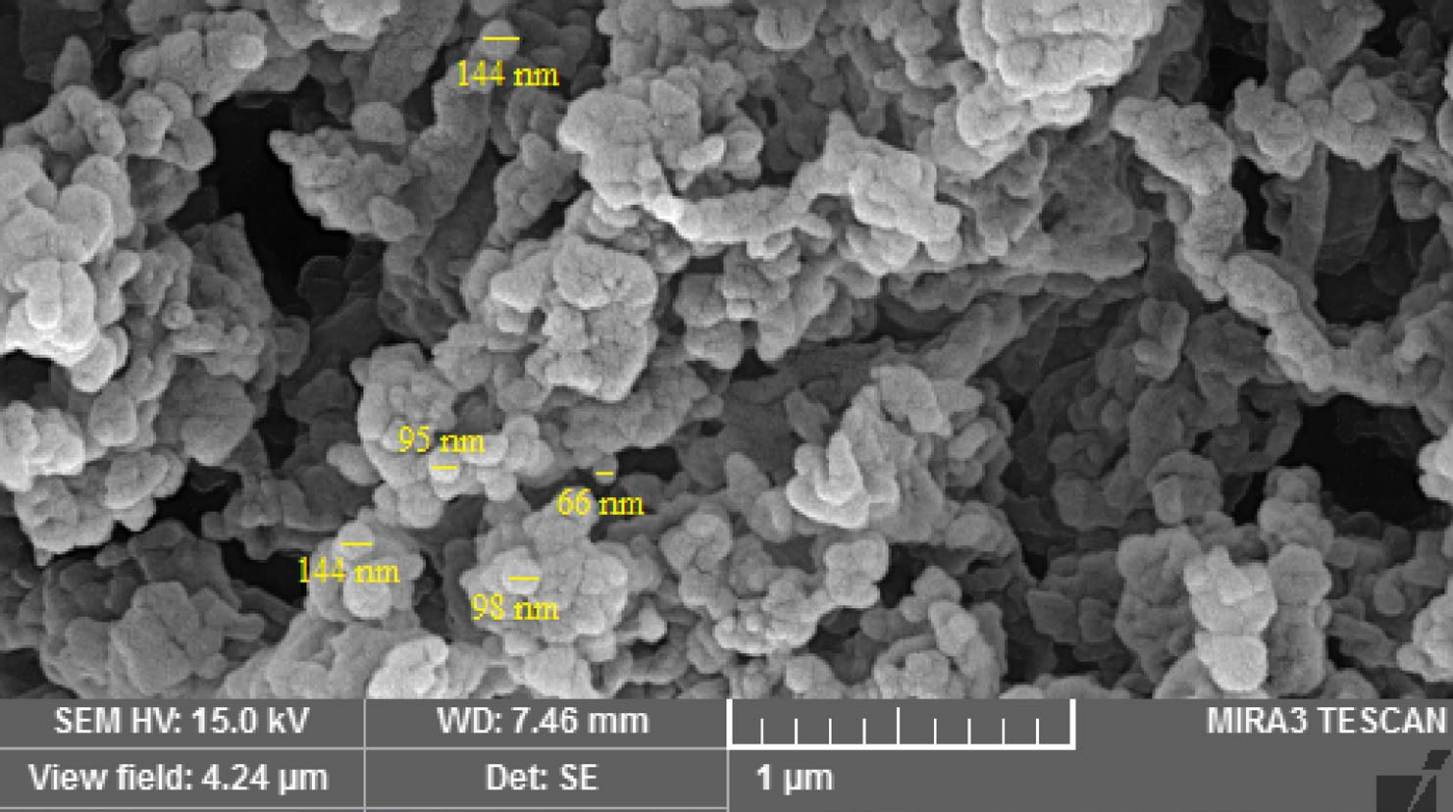
FESEM of CS/rGO/Pa.

SEM results for CS/rGO/Pa exhibited aggregates of the nanocomposites (Fig. 2). The results indicated that the nanocomposites had approximately spherical morphology with an average size of around 100 nm (Fig. 2). The formation of larger aggregates of CS/rGO/Pa nanocomposites (indicated by DLS results), compared to what was observed in SEM images, is likely attributed to electrostatic forces, amphiphilic behavior, and the adsorption of papain molecules, which alter surface properties and promote the particles agglomeration, ultimately leading to an increased hydrodynamic diameter^44^.

### Enzyme activity

Papain enzyme is a protease and it was used in this study for the purpose of debridement and skincare. The results showed that the specific activity of free and immobilized enzyme is 7.3 and 0.57 U/mg, respectively. This result indicates that papain adsorption on CS/rGO, decreased the enzyme activity up to 12.8-fold. It has been shown that the enzyme immobilization can decrease the immobilized enzyme activity due to the increase in the structural rigidity and restricted accessibility to substrates in immobilized enzymes^45^. This observation is consistent with previous studies by Yang et al.^46^, and Zhao et al.^47^, who reported that enzyme immobilization on rGO leads to decreased enzymatic activity. The high activity of papain may have a negative effect on wound healing and skincare and it is necessary to provide an optimal concentration and activity for the enzyme^48,49^. Therefore. the low and gradual enzyme activity in CS/rGO/Pa is more preferable, acceptable and effective for the use in biomedical applications.

### Study of papain release and drug release kinetics

Evaluating the performance of a biomaterial-based delivery system is essential for optimizing both enzyme immobilization efficiency and controlled release in cosmetic and therapeutic applications^50^. In facial masks, precise regulation of enzyme activity enhances skin rejuvenation and targeted treatment, while in biomedical applications, responsiveness to physiological conditions plays a key role^1^. The pH of the skin varies depending on its state for instance, post-inflammatory sites may exhibit alkaline conditions, whereas healthy skin remains mildly acidic^51^. Designing an adaptive system that ensures stability for enzyme immobilization while providing controlled release in skincare applications offers multifunctional benefits.

**Fig. 3:**
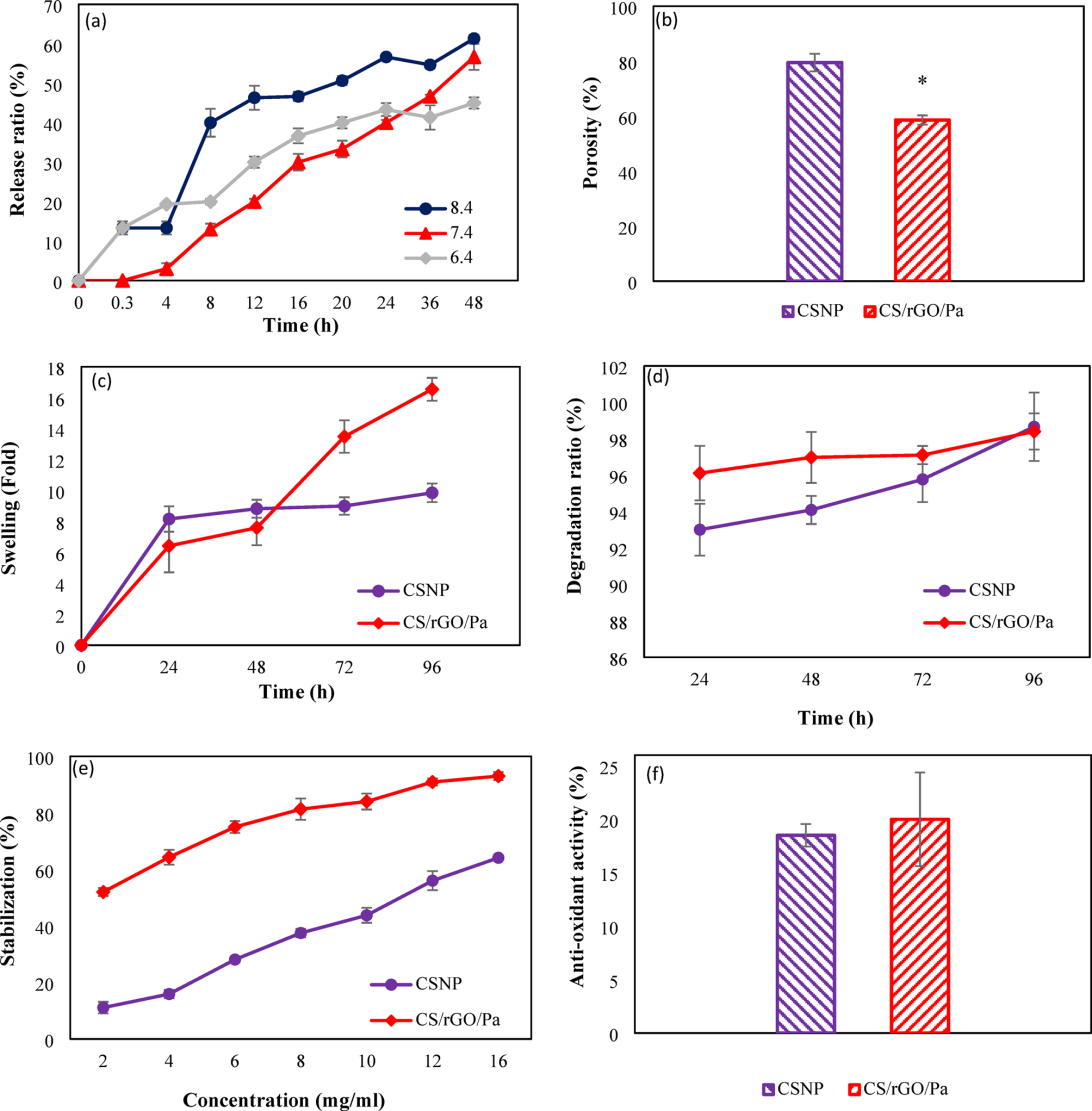
(**a**) Papain release of CS/rGO/Pa nanocomposite during 2 days in three different pHs at 37 °C. (**b**) Porosity study of the CS/rGO/Pa. *indicates a significant decrease in porosity for the sample compared to the control (CSNP) (*p* < 0.05). (**c**) Swelling rate (fold) of the CS/rGO/Pa. (**d**) Degradation rate of the CS/rGO/Pa. (**e**) Anti-inflammatory (stabilization) of the CS/rGO/Pa. (**f**) Anti-oxidant of the CS/rGO/Pa.

According to Fig. 3a, the release rate of papain is an upward trend. The release rate at pH 7.4 experienced a gradual increase during the test period, reaching 56.6%. However, the release rate at pH 6.4 fluctuated modestly, and despite its huge initial release at first 4 h, decreased by 45% at 48 h. In contrast, the release rate at pH 8.4 maintained its bottom-most position, after a surge at 8 h, experienced a moderate increase, and reached 61.3%. At pH 6.4, the chitosan has positive charge and the surface charge of papain is positive due to its isoelectric point (pI 8.75)^38^, while rGO carries a mildly negative charge^52^. This results in minimal electrostatic repulsion and limited enzyme release. In contrast, at pH 8.4, the highly negative charge of rGO^52^, low positive charge of chitosan and approximately neutral charge of papain induces stronger repulsion, facilitating greater enzyme release under alkaline conditions.

The study on papain release kinetics demonstrated stronger correlation coefficients for the Korsmeyer–Peppas model, suggesting that this model primarily dictates the release mechanism of papain from CS/rGO nanocomposites. As shown in Table 2, the release exponent ‘n’ for papain at pH 6.4 and 8.4 was below 0.5, indicating a Fickian diffusion-controlled release. This mechanism suggests that drug (papain) release is primarily governed by diffusion, where molecules move from the delivery system into the surrounding medium due to a concentration gradient. In this case, factors such as the structure of chitosan nanoparticles, the molecular size of papain, and polymer swelling likely contribute to this controlled release behavior^53^. However, at pH 7.4, the release exponent ‘n’ ranged between 0.5 and 1, signifying anomalous (non-Fickian) transport. This mixed mechanism involves both diffusion and polymer relaxation (swelling or erosion), suggesting that multiple transport processes influence papain release under these conditions.

**Table 2:** The calculated correlation coefficients and kinetic parameters for the release of papain from CS/rGO nanocomposites at different pHs.

Sample	pH	Zero order	First order	Higuchi model	Korsmeyer–Peppas model	
		R^2^	R^2^	R^2^	R^2^	n
CS/rGO/Pa	6.4	0.85	0.5	0.95	0.96	0.27
	7.4	0.97	0.6	0.97	0.98	0.87
	8.4	0.83	0.4	0.94	0.96	0.35

### Study of the nanocomposite’s porosity

Porosity is a key structural parameter in nanocomposites, influencing their mechanical strength, and functional performance in biomedical applications^54^. An optimal porosity level is essential; excessive porosity may compromise mechanical integrity, while insufficient porosity can hinder fluid diffusion and cellular infiltration^55^. The methanol displacement test is a reliable method used to determine the porosity. Illustrated in Fig. 3b, the porosity of CSNP was decreased by adding rGO and papain, forming nanocomposite. The porosity of CS/rGO/Pa decreased by 20.9%, reaching 58.6%. The porosity reduction in CS/rGO/Pa related to CSNP might be due to increased intermolecular interactions or enhanced polymer network strength. Ultimately, the porosity required for skincare applications is 50–80%^56,57^, CS/rGO/Pa can be a favorable candidate to use in skincare application.

### Study of the swelling and in vitro degradation

Swelling and degradation behavior play a fundamental role in the performance of nanocomposites, particularly in biomedical applications where material stability and functionality are crucial^58^. The swelling capacity affects water absorption, mechanical properties, and the release of bioactive agents^59^, while the degradation rate determines the material’s lifespan and its interaction with surrounding tissues^60^.

According to Fig. 3c, the swelling rate of CSNP and CS/rGO/Pa follow the same pattern over the first 48 h. However, during the second 48 h, the CS/rGO/Pa experienced a surge, reaching a 16.55-fold increase and the CSNP showed a more modest enhancement, with a 9.85-fold increase. This increase in CS/rGO/Pa swelling is related to hydrophilic rGO and papain. These results are in agreement with the study by Thangavel et al.^61^, which demonstrated that the incorporation of rGO leads to an increase in the swelling capacity.

As shown in Fig. 3d, CSNP and CS/rGO/Pa degraded by around 98% during 96 h. Therefore, CS/rGO/Pa can be a favorable candidate to use in biomedical applications.‍.

### Study of the in vitro anti-inflammatory

The anti-inflammatory potential of the samples was assessed by their ability to stabilize red blood cells (RBCs) against hemolysis in a hypo-saline environment^62,63^. Since excessive hemolysis can trigger inflammatory responses, evaluating RBC membrane stabilization provides valuable insights into the material’s biocompatibility and anti-inflammatory properties^64^. As shown in Fig. 3e, with the presence of rGO and papain the anti-inflammatory activity rosed by 41% related to CSNP (11.1%) at 2 mg/mL concentration, gradually escalated, and reached a peak of 93% at 16 mg/mL concentration. Chitosan is a weak anti-inflammatory material although its composition with rGO and papain which are anti-inflammatory components result in a stronger anti-inflammatory agent. These results are consistent with the findings of Kesavan et al.^22^, who reported that the anti-inflammatory activity increases with the incorporation of rGO into chitosan. To sum up, CS/rGO/Pa might be one of the best candidates for use in facial masks from the anti-inflammatory point of view.

### Anti-oxidant activity

The anti-oxidant activity test was performed using DPPH, investigating the ability of CS/rGO/Pa to scavenge free radicals and inhibit oxidative damage. As indicated in Fig. 3f, the anti-oxidant activity of CS/rGO/Pa is 20%, which is 2% as high as CSNP. Papain is not as strong anti-oxidant agent as rGO^11^. This enhancement corresponds to rGO due to its primary active site, consisting of a sp^2^ carbon network that donates electrons and imparts anti-oxidant activity^61^. The results of this study are consistent with previous reports by Ngo et al.^65^ on the anti-oxidant properties of chitosan, Manosroi et al.^66^ on the anti-oxidant activity of papain, and Vatandost et al.^67^ regarding the anti-oxidant characteristics of rGO. However, in CS/rGO/Pa, the anti-oxidant activity showed a slight increase, which can be explained by correlation with the coating of anti-oxidant groups on rGO by chitosan and papain molecules.

### Anti-bacterial activity

**Fig. 4:**
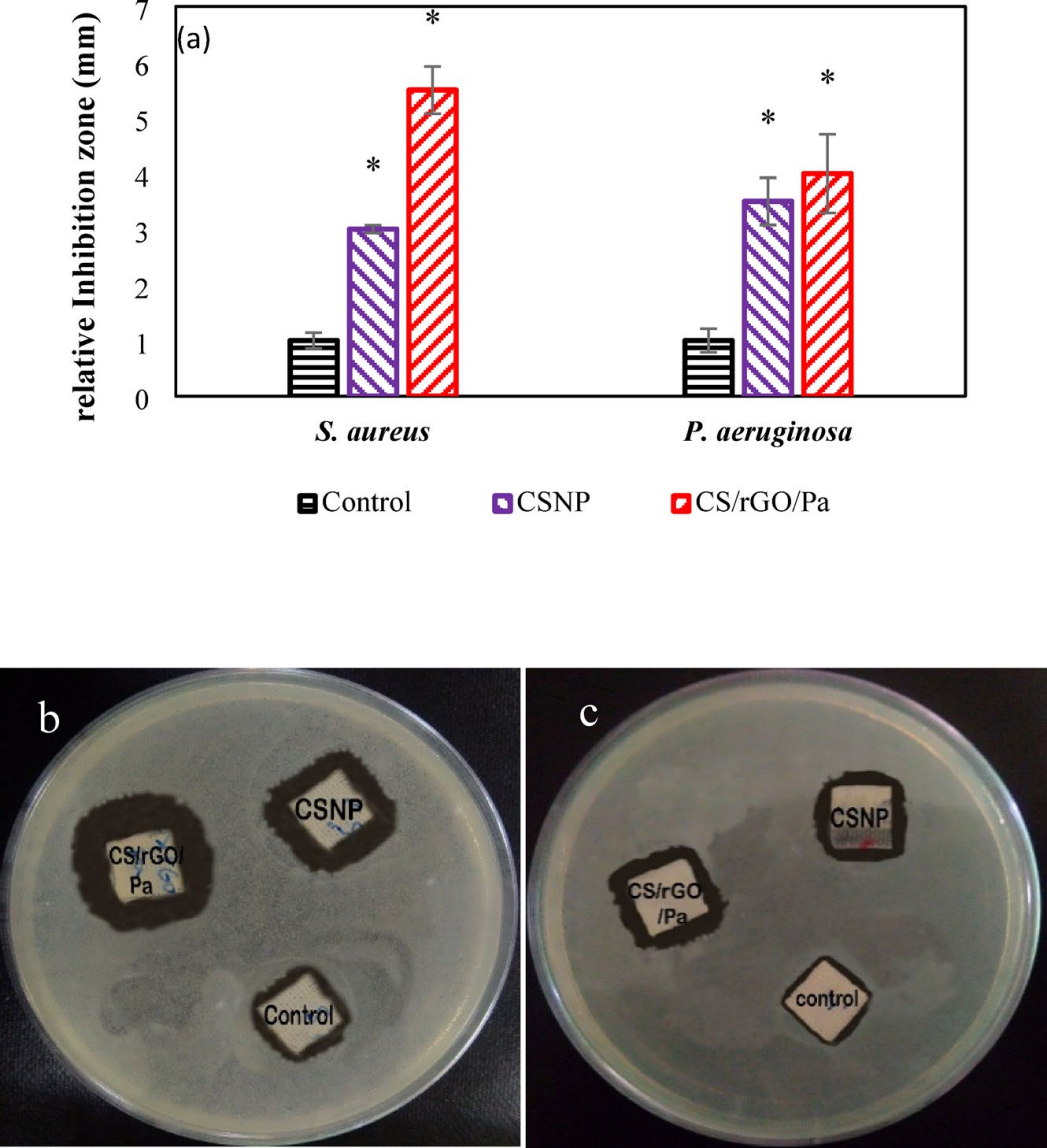
Anti-bacterial properties of samples via disk diffusion method against two skin disease-causing pathogens. (**a**) Relative inhibition zone of samples (compared to cotton). *, ** indicate a significant increase in anti-bacterial capacity when compared with control (cotton) (*p < 0.05, ** *p* < 0.001). Optical images showing the zone of inhibition of the samples against (**b**) *S. aureus*, and (**c**) *P. aeruginosa*, after 18 h of cultivation on agar.

The anti-bacterial test was done against *Staphylococcus aureus* (Gram-positive), and *Pseudomonas aeruginosa* (Gram-negative) on nutrient agar plate over 18 h. Among the bacteria isolated from skin wounds, *S. aureus* accounted for 37% and *P. aeruginosa* for 17% of the total isolates, indicating their high prevalence in wound infections^68^. As shown in Fig. 4a–c, in both bacteria, CS/rGO/Pa indicated better anti-bacterial efficiency. However, this activity is lower against *P. aeruginosa* and the difference between CSNP and CS/rGO/Pa is negligible. Cotton exhibited an inhibition zone of 1 mm due to its intrinsic anti-bacterial activity^24–27^. rGO is a great anti-bacterial agent due to its sharp edge and its ability to damage and rupture the membrane of bacteria^69^. Papain displays anti-bacterial activity owing to its amidase and esterase activities^70^. CSNPs exhibited stronger inhibitory effects against *P. aeruginosa* than *S. aureus*, which aligns with previous reports^71,72^. However, CS/rGO/Pa showed greater efficacy against *S. aureus*. This shift in anti-bacterial pattern may be attributed to the combined oxidative properties of rGO, the proteolytic activity of papain disrupting surface proteins of *S. aureus*, and the potentially higher uptake of nanoparticles by the thicker cell wall of Gram-positive bacteria^73^. These findings are consistent with the study by Mann et al.^69^, which demonstrated that the presence of rGO enhances the anti-bacterial activity of the samples. In general, CS/rGO/Pa can act as a favorable anti-bacterial composite.

### Study of the cell culture and growth

**Fig. 5:**
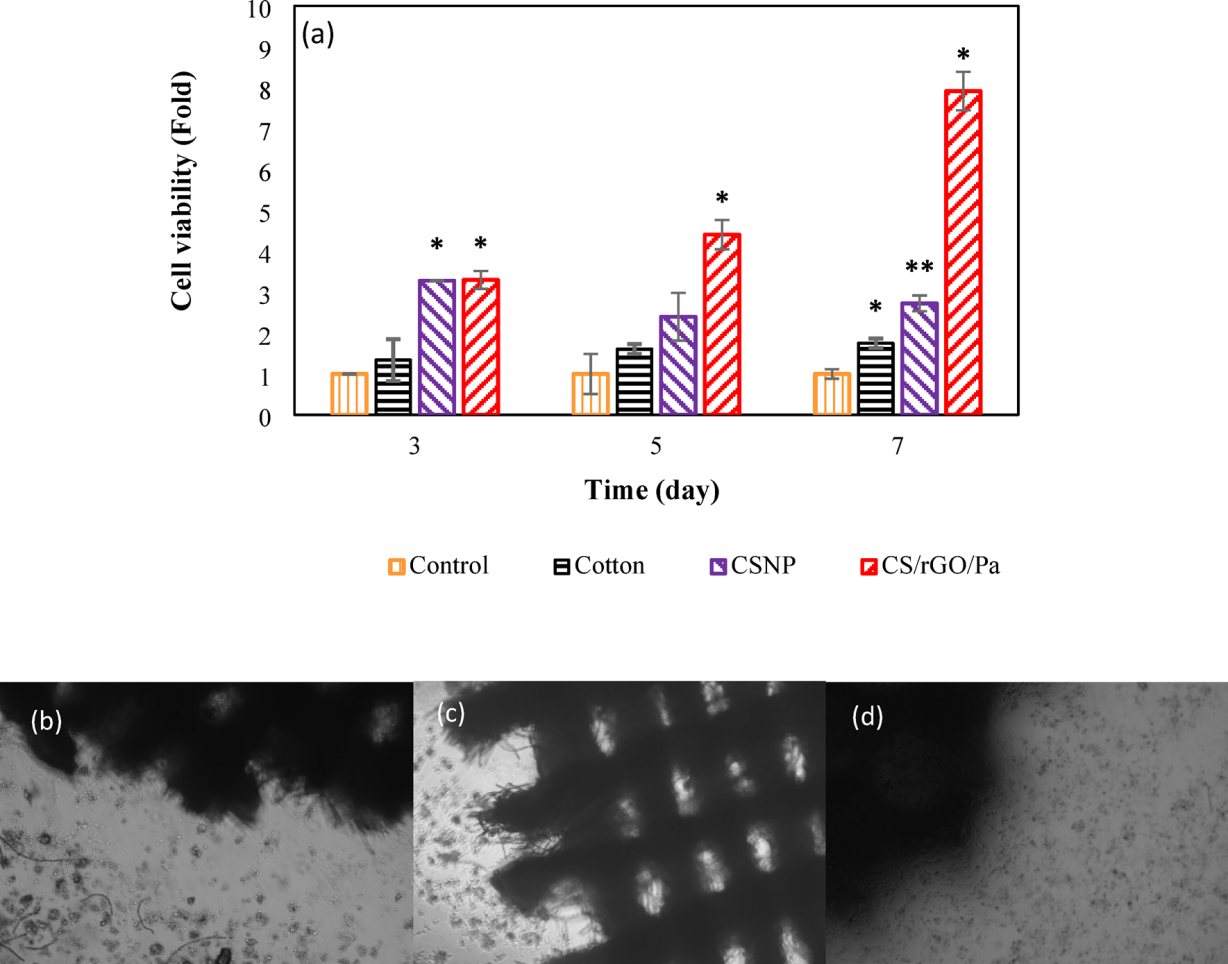
(**a**) Cell viability (fold) of NIH-3T3 fibroblast cells treated against CSNP and CS/rGO/Pa for 7 days. *, ** indicate a significant increase in cell viability when compared with control (*p < 0.05, ** *p* < 0.001). Cell viability (live/dead) images of NIH-3T3 fibroblast cells after the 5th day of treatment against (**b**) Cotton, (**c**) CSNP, and (**d**) CS/rGO/Pa.

Cell viability and growth were assessed using the MTT assay, which evaluates the metabolic activity of cells and provides insights into the effects of the tested materials on cellular growth. According to the results (Fig. 5a–d), no cell death was observed during the test period. The cell growth within CSNP increased gradually, reaching a 2.72-fold increase by day 7. However, in CS/rGO/Pa, the cell proliferation dramatically increased over 7 days, reaching a 7.91-fold increase. Studies have shown that rGO has excellent cell adhesion and proliferation properties and can stimulate angiogenesis by boosting reactive oxygen species (ROS) levels. In addition, rGO can stimulate collagen synthesis and vascularization^74^. Papain’s enzymatic activity can promote cell growth by reducing inflammation and oxidative stress^75^. Therefore, this sample is an appropriate material from the cell growth point of view.

## Conclusion

This study successfully developed a multifunctional bioactive nanocomposite based on chitosan, reduced graphene oxide, and papain, tailored for cosmetic and biomedical applications. The formulation was successfully prepared using the ion gelation method and characterized through FTIR, XRD, SEM and DLS analyses, confirming the proper incorporation of each component. Notably, the controlled immobilization of papain on the CS/rGO matrix led to a moderated enzymatic activity, which may enhance its long-term stability and performance in topical applications. In vitro evaluations revealed that the nanocomposite exhibited notable synergistic effects compared to its individual components, as evidenced by enhanced anti-inflammatory, anti-oxidant and anti-bacterial activities, along with favorable cytocompatibility. In addition, the addition of rGO within the chitosan matrix improved the surface hydrophilicity of the nanocomposite, which is advantageous for cell attachment and topical applications. The structural stability of the CS/rGO matrix also contributed to sustained functional performance during in vitro evaluations, indicating its potential for stable and reliable bioactive delivery. The study highlights the potency of papain when adsorbed on CS/rGO for application in different biomedical use such as wound healing and cosmetic facial masks. Looking ahead, this platform opens new avenues for the design of smart wound dressings and next-generation facial masks with sustained bioactivity. Future studies may explore: (1) Formulation optimization for commercial cosmetic products targeting sensitive or aging skin. (2) Integration with other bioactive agents (e.g., growth factors, peptides) to enhance tissue remodeling. (3) Responsive delivery systems that adapt to skin microenvironment changes (e.g., pH, temperature, inflammation). By bridging nanotechnology and dermatological science, this work lays the foundation for advanced skin-care solutions and regenerative therapies with real-world clinical potential.

## Data Availability

Data will be available on request.
